# Fermentation by *Lactobacillus *enhances anti-inflammatory effect of Oyaksungisan on LPS-stimulated RAW 264.7 mouse macrophage cells

**DOI:** 10.1186/1472-6882-12-17

**Published:** 2012-03-12

**Authors:** You-Chang Oh, Won-Kyung Cho, Jin Hui Oh, Ga Young Im, Yun Hee Jeong, Min Cheol Yang, Jin Yeul Ma

**Affiliations:** 1 Traditional Korean Medicine (TKM)-Based Herbal Drug Research Group, Korea Institute of Oriental Medicine, Yuseong, Daejeon, Republic of Korea; 2 Traditional Korean Medicine (TKM)-Based Herbal Drug Research Group, Korea Institute of Oriental Medicine, 461-24, Jeonmin-dong, Yuseong, Daejeon 305-811, Republic of Korea

## Abstract

**Background:**

Oyaksungisan (OY) has been used as a traditional drug in east-Asian countries. However, its effect on inflammation still remains unknown. In this study, to provide insight into the biological effects of OY and OY fermented by *Lactobacillus*, we investigated their effects on lipopolysaccharide (LPS)-mediated inflammation in the RAW 264.7 murine macrophage cells.

**Methods:**

The investigation was focused on whether OY and fermented OYs could inhibit the production of pro-inflammatory mediators such as nitric oxide (NO) and prostaglandin (PG) E_2 _as well as the expression of inducible nitric oxide synthase (iNOS), cyclooxygenase (COX)-2, tumor necrosis factor (TNF)-α, interleukin (IL)-6, nuclear factor (NF)-κB and mitogen-activated protein kinases (MAPKs) in LPS-stimulated RAW 264.7 cells.

**Results:**

We found that OY inhibits a little LPS-induced NO, PGE_2_, TNF-α and IL-6 productions as well as the expressions of iNOS and COX-2. Interestingly, the fermentation significantly increased its inhibitory effect on the expression of all pro-inflammatory mediators. Furthermore, the fermented OYs exhibited elevated inhibition on the translocation of NF-κB p65 through reduced IκBα degradation as well as the phosphorylations of extracellular signal-regulated kinase (ERK), p38 and c-Jun NH_2_-terminal kinase (JNK) MAPKs than untreated control or original OY.

**Conclusions:**

Finally, the fermentation by *Lactobacillus *potentiates the anti-inflammatory effect of OY by inhibiting NF-κB and MAPK activity in the macrophage cells.

## Background

Oyaksungisan (OY) is a traditional herbal medication that consists of twelve herbs and is known to have anti-arthralgia, anti-paralysis and anti-dizziness effects. Since ancient times, OY has been widely used as a traditional medication in Asian countries. More specifically, it has been prescribed for the treatment of beriberi, vomiting, diarrhea and circulatory disturbance.

Recent studies have demonstrated that OY inhibits the adjuvant arthritis in rat [1] and OY has neuroprotective activity [2]. It was also reported that OY exerts the protective effect against H_2_O_2_-induced apoptosis [3] and other studies have revealed the anti-inflammatory effect in peripheral blood mononuclear cells from cerebral infarction patients [4]. However, the effect and mechanism of OY or fermented OYs on macrophage-mediated inflammation still remain unknown.

The fermented plant products are abundant in antioxidants similar to butylated hydroxyanisole and green tea [5]. During the process of fermentation by *Lactobacillus*, organic acids are amassed, proteins are hydrolyzed and antioxidant ferulic acid from plant cell wall materials are solubilized [6,7]. When the grain foods once were fermented with *Aspergillus oryzae*, strong antioxidant destroying free radicals were formed [8]. Those fermented plant products could contain antioxidative activities and anti-inflammatory activities, which can be used as a good and alternative method to treat patients who suffer from diseases like cancer and HIV [9].

Inflammation is an early host immune reaction mediated by cytokines secreted from immune cells. Various *in vitro *and *in vivo *experimental models have been established to assess the inhibitory effects of naturally derived products on the synthesis and release of inflammatory cytokines and other inflammatory mediators including NO and PGE_2_, which are synthesized by iNOS and COX-2, respectively [10,11]. LPS is one of well-known inflammatory ligands to stimulate macrophages to release various inflammatory cytokines. These inflammatory cytokines are essential for host survival following infection and are also required for the repair of tissue injuries [11]. Of these pro-inflammatory cytokines, TNF-α and IL-6 are known to be important inflammatory mediators involved in the development of a number of inflammatory diseases.

Macrophages play an important role in host defenses against noxious substances and are involved in a variety of disease processes including autoimmune diseases, infections, and inflammatory disorders [12]. Various inflammatory mediators are involved in the pathogenesis of many inflammation-associated human diseases [13,14]. The expression of these cytokine genes can be regulated by the activation of the NF-κB, which is critically involved in the pathogenesis of rheumatoid arthritis and other chronic inflammatory diseases [15]. Usually, p65 of NF-κB is tightly sequestered by IκBα in the cytosol but when immunostimulatory ligands like LPS activate the cells, p65 is released from the phosphorylated IκBα by IκBα kinase and subsequent IκBα degradation [16]. The liberated NF-κB then translocates from cytosol into nuclei and transactivates the promoter of pro-inflammatory genes such as iNOS, COX-2 and IL-6. Most anti-inflammatory drugs suppress the expression of these pro-inflammatory genes by inhibiting the NF-κB activation pathway [17].

In addition, MAPKs phosphorylation is related to induce NF-κB activation and to stimulate iNOS gene expression. Several reports showed specific MAPK inhibitors suppress iNOS gene expression [18-20].

Here, we first demonstrate the inhibitory effect of OY and fermented OYs on LPS-induced inflammation in RAW 264.7 macrophage cells by evaluating the expression of pro-inflammatory mediators and furthermore, OY fermented by *Lactobacillus *exhibit strong anti-inflammatory activity by repressing pro-inflammatory mediators via regulating NF-κB and MAPK signaling.

## Methods

### Materials and reagents

RPMI 1640, penicillin, and streptomycin were obtained from Hyclone (Logan, UT, USA). Bovine serum albumin, LPS and 3-(4,5-dimethylthiazol-2-yl)-2,5-diphenylthiazolium bromide (MTT) were purchased from Sigma (St. Louis, MO, USA). COX-2, iNOS antibodies were purchased from Abcam (Cambridge, UK). β-actin, p38, phospho-p38, ERK, phospho-ERK, JNK, phospho-JNK and p65 monoclonal antibodies were purchased from cell signaling technology, Inc. (Boston, MA, USA). Peroxidase-conjugated secondary antibodies were obtained from Santa Cruz Biotechnology Inc. (Santa Cruz, CA, USA) and TNF-α, IL-6 antibody, biotinylated TNF-α and IL-6 antibodies were purchased from BD Biosciences (San Jose, CA, USA). An RNA extraction kit was purchased from iNtRON Biotech (Daejeon, Korea). The primers specific for COX-2, iNOS, IL-6, and β-actin were synthesized by Bioneer Corp. (Daejeon, Korea). Poncirin was obtained from the Korea Food & Drug Administration (Cheongwon, Korea) and Hesperidin was purchased from ICN Co. (Costa Mesa, CA, USA). Glycyrrhizin was purchased from TCI Co. (Tokyo, Japan) and Rutin and Naringin were purchased from Sigma Chemical Co. (St. Louis, MO, USA). HPLC grade solutions, water, acetonitrile, and glacial acetic acid were purchased from J.T. Baker (Austin, TX, USA).

### Preparation of OY and fermented OYs

The OY is composed of Ephedra Herb, Citrus Unshiu Peel, Lindera Root, Cnidii Rhizoma, Angelica Dahurica Root, Batryticatus Bombyx, Aurantii Fructus Immaturus, Platycodon Root, Zingiberis Rhizoma, Glycyrrhizae Radix et Rhizoma, Zingiberis Rhizoma Crudus, Zizyphi Fructus, which were purchased from Yeongcheon Oriental Herbal Market (Yeongcheon, Korea). All voucher specimens were deposited in the herbal bank and placed in 22,345 mL of distilled water and then extracted by heating for 3 hours at 115°C (Gyeongseo Extractor Cosmos-600, Inchon, Korea). After extraction, the OY was filtered out using standard testing sieves (150 μm) (Retsch, Haan, Germany). The OY was incubated with *Lactobacilluses *(1-5 × 10^8 ^CFU/mL) obtained from the KFRI (Korea Food Research Institute, Sungnam, Korea) to prepare fermented OYs. Before use, the bacterial strain was incubated in 50 mL of MRS broth (DifcoTM Lactobacilli MRS Broth, Becton Dickinson, Franklin Lakes, NJ, USA) at 37°C overnight. The OY fermented by *Lactobacilluses *at 37°C for 48 hours was filtered with a 60-μm nylon net filter (Millipore, Billerica, MA, USA), lyophilized, and stored in desiccators at 4°C. The freeze-dried extract powder was then dissolved in phosphate buffered saline (PBS) and filtered (pore size, 0.45 μm), lyophilized and kept at 4°C prior to use.

### Cell culture

The murine macrophage cell RAW 264.7 was obtained from the Korea Cell Line Bank (Seoul, Korea) and grown in RPMI 1640 medium containing 10% fetal bovine serum and 100 U/mL of penicillin/streptomycin sulfate. The cells were incubated in a humidified 5% CO_2 _atmosphere at 37°C [21]. To stimulate the cells, the medium was replaced with fresh RPMI 1640 medium with 200 ng/mL LPS in the presence or absence of OY and fermented OYs for the indicated periods.

### Methylthiazolyl tetrazolium (MTT) assay for cell viability

Cytotoxicity was analyzed using an MTT assay. RAW 264.7 cells were seeded at 5 × 10^4 ^cells/mL densities in 96-well plates (Nunc, Roskilde, Denmark). Each group had a nontreated group as a control. OY or fermented OYs (10, 100, 500 and 1000 μg/mL) was added to each well and incubated for 48 hours at 37°C and 5% CO_2_. The MTT solutions (5 mg/mL) were added to each well and the cells were cultured for another 4 hours. The supernatant was then discarded and 100 μL of dimethyl sulfoxide (DMSO) was added to each well. The absorbance at 570 nm was read using ELISA reader.

### Measurement of NO production

NO production was analyzed by measuring the nitrite formed in the supernatants of cultured RAW 264.7 cells. The cells were seeded at 5 × 10^5 ^cells/mL in 96-well culture plates. After preincubation of the RAW 264.7 cells for 18 hours, the cells were pretreated with OY or fermented OYs (10, 100 and 500 μg/mL) and stimulated with LPS (200 ng/mL) for 24 hours. The supernatant was mixed with an equal volume of Griess reagent (1% sulfanilamide, 0.1% naphthylethylenediamine dihydrochloride, and 2.5% phosphoric acid) and incubated at room temperature for 5 min. The concentrations of nitrite were measured by reading at 570 nm. Sodium nitrite (NaNO_2_) was used to generate a standard curve.

### Measurement of PGE_2 _production

The RAW 264.7 cells were cultured in 24-well culture plates (5 × 10^5 ^cells/mL). The cells were pretreated with OY or fermented OYs (10, 100 and 500 μg/mL) and stimulated with LPS (200 ng/mL) for 24 hours. The supernatant was collected for PGE_2 _determination by a PGE_2 _Express EIA Kit (Cayman chemical, Ann Arbor) according to the manufacturer's instructions.

### Enzyme-linked immunosorbent assay (ELISA)

Cells were seeded at 5 × 10^5 ^cells/mL per well in 24-well culture plates and pretreated with various concentrations of OY or fermented OYs (10, 100 and 500 μg/mL) for 30 min before LPS stimulation (200 ng/mL). ELISA plates (Nunc, Roskilde, Denmark) were coated overnight at 4°C with anti-mouse TNF-α or IL-6 antibodies diluted in coating buffer (0.1 M carbonate, pH 9.5) and then washed four times with PBS containing 0.05% Tween 20. The nonspecific protein binding sites were blocked with assay diluent (PBS containing 10% fetal bovine serum, pH 7.0) for at least 1 hour. Immediately, each samples and standards were added to the wells. After incubation for 2 hours, a working detector (biotinylated anti-mouse TNF-α or IL-6 monoclonal antibody and streptavidin-horseradish peroxidase reagent) was added and incubated for 1 hour. Subsequently, substrate solution (tetramethylbenzidine) was added to the wells and incubated for 30 min in the dark until the reaction was stopped with stop solution (2 N H_3_PO_4_). The absorbance at 450 nm was read. All subsequent steps took place at room temperature and all standards and samples were assayed in duplicate.

### Western blot analysis

Protein expression was assessed by Western blot analysis according to standard procedures. The RAW 264.7 cells were cultured in 60-mm-diameter culture dishes (1.5 × 10^6 ^cells/mL) and pretreated with OY or fermented OYs (500 μg/mL). After 30 min, LPS (200 ng/mL) was added to the cells and the cells were incubated at 37°C for the indicated periods. After incubation, the cells were washed twice in ice-cold PBS (pH 7.4). The cells were resuspended in lysis buffer containing 50 mM Tris-base (pH 7.5), 150 mM NaCl, 2 mM EDTA, 1% glycerol, 10 mM NaF, 10 mM Na-pyrophosphate, 1% NP-40 and protease inhibitors (0.1 mM phenylmethylsulfonylfluoride, 5 μg/mL aprotinin, and 5 μg/mL leupeptin) on ice for 15 min, and cell debris was removed by centrifugation. The protein concentrations were determined using the Bio-Rad protein assay reagent (Bio-Rad Laboratories, Hercules, CA, USA) according to the manufacturer's instructions. Equal amounts of protein (20 μg) were subjected to sodium dodecyl sulfate- polyacrylamide gel electrophoresis (SDS-PAGE) and then transferred onto a polyvinylidene membrane (Millipore, Bedford, MA, USA). The membrane was blocked with 5% nonfat milk in Tris-buffered saline (150 mM NaCl, 20 mM Tris-HCl, pH 7.4) with 0.05% Tween 20 buffer. After blocking, the membrane was incubated with primary antibodies against COX-2, iNOS, ERK, phospho-ERK, p38, phospho-p38, JNK and phospho-JNK for 18 hours at 4°C. The membrane was then washed with Tris-buffered saline containing Tween 20 and incubated with anti-mouse or anti-rabbit immunoglobulin G horseradish peroxidase (HRP)-conjugated secondary antibodies. The specific proteins were detected using enhanced chemiluminescence (ECL) (Millipore, Billerica, MA, USA).

### Preparation of cytosolic and nuclear proteins for IκBα and NF-κB

The RAW 264.7 cells pretreated with OY or fermented OYs were stimulated with LPS (200 ng/mL). For the detection of IκBα, cytosolic fractions were prepared as follows. The cells were washed twice in cold PBS, incubated on ice for 10 min in lysis buffer [25 mmol/L Tris-HCl (pH 7.4), 150 mmol/L NaCl, 1 mmol/L CaCl_2_, 1% Triton X-100, 1 mmol/L phenylmethylsulfonyl fluoride, and 10 μL/mL aprotinin], and centrifuged at 15,000 g for 10 min at 4°C. To prepare the nuclear fractions, the cells were washed with 1 mL of ice-cold PBS, resuspended in 400 μL of ice-cold hypotonic buffer [10 mmol/L HEPES/KOH, 10 mmol/L KCl, 2 mmol/L MgCl_2_, 0.1 mmol/L EDTA, 1 mmol/L dithiothreitol, and 0.5 nmol/L phenylmethylsulfonyl fluoride (pH 7.9)], left on ice for 10 min, vortex-mixed, and centrifuged at 15,000 g for 30 sec. The pellets were resuspended in 50 μL of ice-cold saline buffer [50 mmol/L HEPES/KOH, 50 mmol/L KCl, 1 mmol/L dithiothreitol, 300 mmol/L NaCl, 0.1 mmol/L EDTA, 10% glycerol, and 0.5 mmol/L phenylmethylsulfonyl fluoride (pH 7.9)], left on ice for 20 min, vortex-mixed, and centrifuged at 15,000 g for 5 min at 4°C to collect the supernatant containing the nuclear fractions. The cytosolic and nuclear fractions were used to detect IκBα or NF-κB via Western blot analysis as described previously.

### RNA extraction and reverse transcription-polymerase chain reaction (RT-PCR)

Total cellular RNA was isolated by the easy-BLUE™ RNA extraction kit (iNtRON Biotech) according to the manufacturer's instructions. The total RNA (2 μg) was converted to cDNA using 200 units of reverse transcriptase and 500 ng of oligo(dT) primer in 50 mM Tris-HCl (pH 8.3), 75 mM KCl, 3 mM MgCl_2_, 10 mM dithiothreitol, and 1 mM deoxynucleotide triphosphates at 42°C for 1 hour. The reaction was stopped by heating at 70°C for 15 min, and the cDNA mixture (3 μL) was used for enzymatic amplification. PCR was performed in 50 mM KCl, 10 mM Tris-HCl (pH 8.3), 1.5 mM MgCl_2_, 0.2 mM deoxynucleotide triphosphates, 2.5 units of Taq DNA polymerase, and 0.1 μM each TNF-α, IL-6, COX-2, iNOS, and β-actin primers, respectively. PCR for TNF-α and COX-2 was conducted by subjecting the reaction mixtures to initial denaturation at 94°C for 5 min and followed by 35 cycles of 94°C for 30 sec, 65°C (TNF-α), 50°C (COX-2) for 30 sec and 72°C for 1 min, with a final extension at 72°C for 7 min [22]. PCR for IL-6 was conducted by heating the reaction mixtures to 94°C for 3 min, after which they were subjected to 35 cycles of 93°C for 45 sec, 57°C for 45 sec and 72°C for 90 sec, with a final extension at 72°C for 7 min [23]. PCR for iNOS was conducted by heating the reaction mixtures to 94°C for 5 min, after which they were subjected to 35 cycles of 94°C for 30 sec, 60°C for 30 sec and 72°C for 1 min, with a final extension at 72°C for 7 min [24]. PCR products were electrophoresed on 1.5% agarose gel and stained with ethidium bromide. The primer sequences are listed in Table 1.

**Table 1:** Primers used for RT-PCR

Target gene	primer sequence
TNF-α	F: 5'-AGCCCACGTCGTAGCAAACCACCAA-3'
	R: 5'-AACACCCATTCCCTTCACAGAGCAAT-3'
IL-6	F: 5'-CATGTTCTCTGGGAAATCGTGG-3'
	R: 5'-AACGCACTAGGTTTGCCGAGTA-3'
COX-2	F: 5'-TTTGATTAGTACTGTAGGGTTAATG-3'
	R: 5'-TTTGATTAGTACTGTAGGGTTAATG-3'
iNOS	F: 5'-CCTCCTCCACCCTACCAAGT-3'
	R: 5'-CACCCAAAGTGCTTCAGTCA-3'
β-actin	F: 5'-TGGAATCCTGTGGCATCCATGAAA-3'
	R: 5'-TAAAACGCAGCTCAGTAACAGTCCG-3'

F, forward; R, reverse

### High-performance liquid chromatography analysis and sample preparation

The analytical HPLC system (Hitachi Co., Tokyo, Japan) consisted of a pump (L-2130), an autosampler (L-2200), a column oven (L-2350) and a diode array UV/VIS detector (L-2455). All operations and data analyses were controlled using Hitachi EZchrom Elite software. The separation of OY, OY-A and OY-B were carried out at 40°C on a J'sphere ODS-H80 column (4.6 mm × 250 mm, 4 μm, YMC Co., Ltd., Kyoto, Japan). The mobile phases consisted of 0.1% acetic acid in deionized water (A) and 0.1% acetic acid in acetonitrile (B) using a gradient elution of 10-10% (v/v) B at 0-10 min; 10-100% B at 10-60 min; 100-100% B at 60-70 min. The flow rate was 1.0 mL/min and the injection volume of samples was 10 μL. Components were identified via comparison of their retention times to those of authentic standards under identical analysis conditions and UV spectra with an in-house PDA-library. The standard stock solutions of poncirin, hesperidin, glycyrrhizin, rutin and naringin were prepared in methanol at a concentration of 100 μg/mL. OY, OY-A and OY-B powder was weighed and dissolved with deionized water at a concentration of 20 mg/mL. Prior to analysis, all solutions were maintained at 4°C and the samples were filtered through a 0.45 μm filter.

### Statistical analysis

The results were expressed as means ± SE values for the number of experiments. Statistical significance was determined between treated group and the control and was calculated by Student's t tests. Each experiment was repeated at least three times to yield comparable results. Values of P < 0.05 and P < 0.005 were considered significant.

## Results

### Effect of OY and fermented OYs on NO and PGE_2 _productions by LPS stimulation

**Figure 1:**
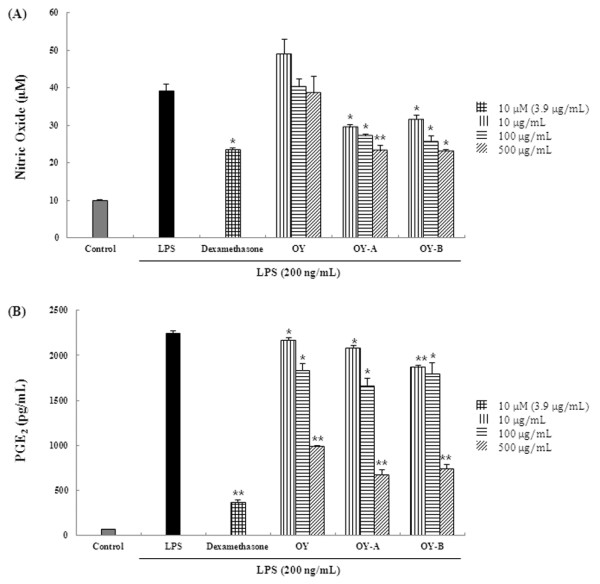
**Effect of OY and fermented OYs on LPS-induced (A) NO and (B) PGE_2 _production**. RAW 264.7 cells pretreated with the indicated each concentration of OY or fermented OYs for 30 min before being incubated with LPS (200 ng/mL) for 24 hours. The culture supernatant was analyzed for nitrite production. The data are mean ± SE values of duplicate determinations from three separate experiments. *p < 0.05 and **p < 0.005, compared with LPS-stimulated values.

First of all, to investigate the anti-inflammatory effect of OY and fermented OYs, we examined the effect of OY and fermented OYs on the NO or PGE_2 _production by LPS stimulation in RAW 264.7 cells. The cells were pretreated with OY or fermented OYs in different concentrations before LPS stimulation and subjected to measure NO production. As a positive control, we used 10 μM dexamethasone, which is used for anti-inflammatory agent. As shown in Figure 1A, non-fermented original form, OY did not show any inhibitory effect on NO production at any concentration but its fermented forms, both OY-A and OY-B at a concentration of 10 μg/mL inhibited more than 25% of NO production and their inhibitory effects were increased dose-dependently. At a concentration of 500 μg/mL, fermented OYs reduced NO production up to 40%. In addition, when LPS-stimulated PGE_2 _secretion was compared in the presence of OY or fermented OYs, OY and fermented OYs at a low concentration showed a little inhibitory effect but fermented OYs repressed more significantly PGE_2 _secretion at a high concentration (500 μg/mL) (Figure 1B).

### The fermented OYs suppress TNF-α and IL-6 expression in LPS-stimulated RAW 264.7 cells

**Figure 2:**
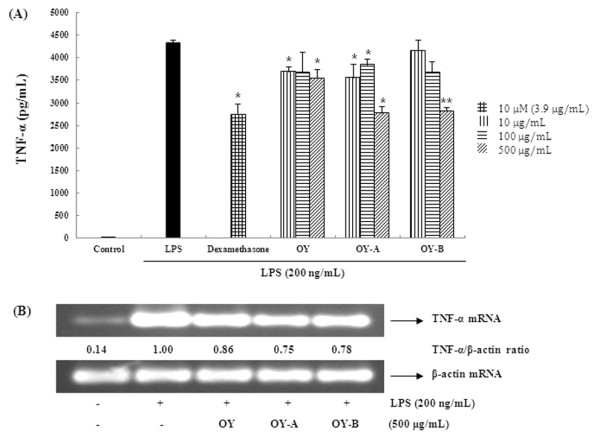
**Effect of OY and fermented OYs on LPS-induced (A) TNF-α cytokine and (B) mRNA expression**. RAW 264.7 cells pretreated with the indicated concentrations of OY or fermented OYs for 30 min before being incubated with LPS (200 ng/mL) for (**A**) 24 hours and (**B**) 6 hours, respectively. The level of TNF-α was measured by ELISA and the mRNA level was assessed by RT-PCR. (**A**) The data are mean ± SE values of duplicate determinations from three independent experiments. *p < 0.05 and **p < 0.005, were calculated from comparing with LPS-stimulated values. (**B**) The values in the LPS-stimulated cells without OY treatment was set to 1.0 and the differences by treatment were quantitated. The experiments were repeated at least three times, and similar results were obtained.

**Figure 3:**
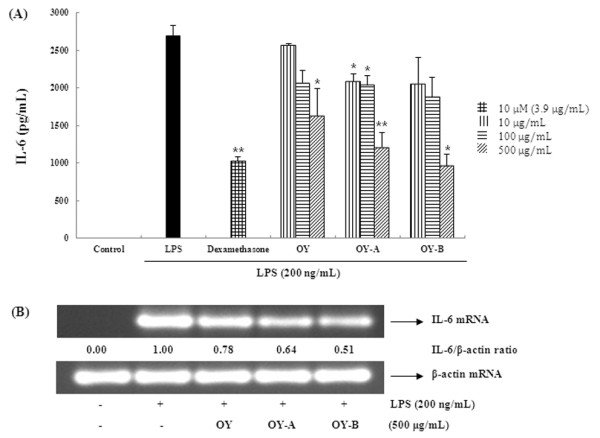
**Effect of OY and fermented OYs on LPS-induced (A) IL-6 cytokine and (B) mRNA expression**. RAW 264.7 cells pretreated with the indicated each concentrations of OY or fermented OYs for 30 min were stimulated with LPS (200 ng/mL) for (**A**) 24 hours and (**B**) 6 hours, respectively. The cytokine and mRNA levels were evaluated by same methods as described in Figure 2.

Since original OY showed little effect on NO and PGE_2 _production but fermented OYs exhibited elevated inhibitory effect on them, we further investigated the effect of OY and fermented OYs on LPS-stimulated TNF-α and IL-6 production using ELISA and RT-PCR. The cells were pretreated with OY or fermented OYs in various concentrations and subjected to measure the levels of TNF-α and IL-6 induced by LPS treatment. Likewise, we used 10 μM dexamethasone as a positive control. As shown in Figure 2A, non-fermented OY showed slight inhibitory effect on TNF-α production but in the presence of 500 μg/mL fermented OY-A or OY-B, TNF-α cytokine production was reduced more than 35% compared to untreated control. Next, we examined the effect of OY and fermented OYs on TNF-α gene expression. The fermented OYs showed much elevated suppressive effect on TNF-α mRNA expression than original OY (Figure 2B). We checked other cytokine IL-6 level in the presence of OY or fermented OYs. As shown in Figure 3A, consistent with TNF-α result, fermented OY-A and OY-B at a concentration of 500 μg/mL inhibited IL-6 cytokine production more than 51% and 61%, respectively. Also, fermented OYs showed more significantly inhibited IL-6 mRNA expression than original OY (Figure 3B). These results suggest OY modified by fermentation exhibits elevated inhibitory activity on the inflammatory cytokine expression in transcriptional level.

### The OY and fermented OYs inhibit LPS-stimulated COX-2 and iNOS expressions

**Figure 4:**
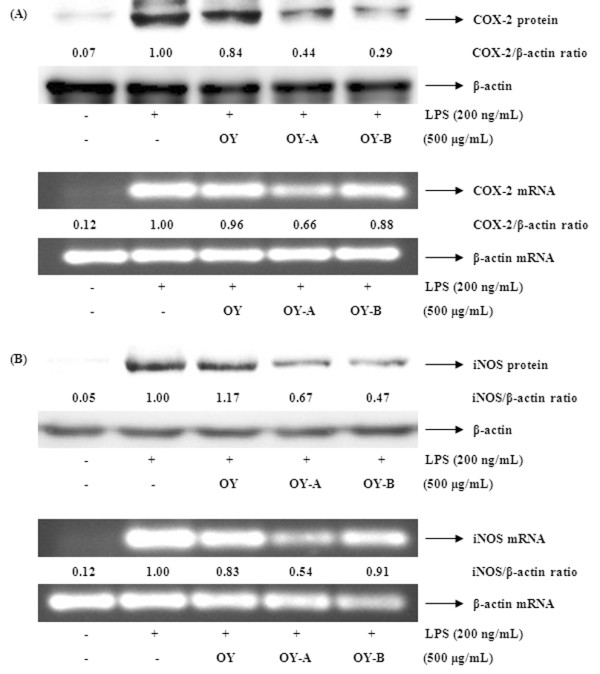
**Effect of OY and fermented OYs on LPS-induced (A) COX-2 and (B) iNOS expression**. RAW 264.7 cells pretreated with OY or fermented OYs (500 μg/mL) for 30 min before being incubated with LPS (200 ng/mL) for 24 hours. Equal amounts of protein were used for immunoblot analysis for detection of COX-2 and iNOS. RT-PCR was used for COX-2 and iNOS mRNAs analysis.

Next, we investigated to determine whether the inhibitory effects of fermented OYs on PGE_2 _and NO production are related to the modulation of their synthesizing enzymes COX-2 and iNOS expression. Usually, in unstimulated cells, COX-2 and iNOS proteins are undetectable, but in response to immunostimulators like LPS, COX-2 and iNOS genes are strongly induced. As shown in Figure 4A, OY showed weak inhibitory effect on both protein and mRNA expression of COX-2 but fermented OYs strongly suppressed COX-2 protein and mRNA expression at a concentration of 500 μg/mL. Also, when iNOS expression was compared in the presence of OY or fermented OYs, consistent with COX-2 results, the fermented OYs, both OY-A and OY-B strongly inhibited iNOS protein and mRNA expression (Figure 4B). These results indicate fermented OYs repress both COX-2 and iNOS gene expression in mRNA and protein levels, thereby inhibiting NO and PGE_2 _production.

### Inhibitory effect of OY and fermented OYs on LPS-induced NF-κB nuclear translocation and IκBα degradation

**Figure 5:**
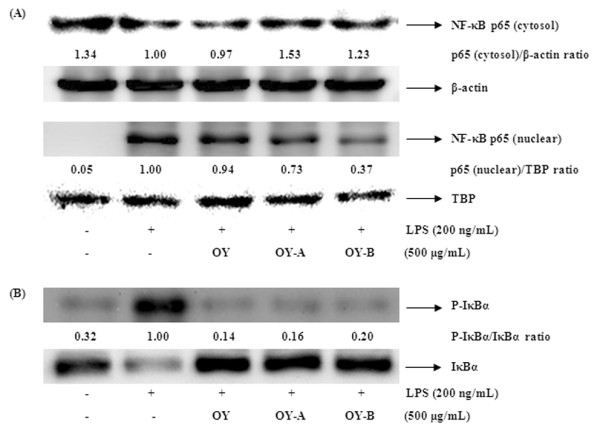
**Effect of OY and fermented OYs on LPS-induced (A) NF-κB translocation and (B) IκBα degradation**. The cells treated with LPS (200 ng/mL) alone or with LPS and OY and fermented OYs (500 μg/mL) for 30 min (IκBα) or for 1 hour (NF-κB). The levels of IκBα and NF-κB (p65) in the nuclear and cytosol extracts were determined by Western blot analysis.

The activation of NF-κB is necessary for the induction of the iNOS and COX-2 genes. Since p65 translocation is an indicator of NF-κB activation by LPS stimulation, we checked the protein levels of p65 in the nuclear and cytoplasmic fractions using Western blot analysis. As shown in Figure 5A, the LPS-induced nuclear translocation of p65 was a little inhibited by non-fermented OY, but fermented OYs significantly increased the inhibitory effect on nuclear translocation of p65 at a concentration of 500 μg/mL. TATA box-binding protein (TBP) was used as a loading control in the nuclear protein. Since the nuclear translocation of p65 is occurred by IκBα phosphorylation and degradation, we also examined the cytoplasmic levels of IκBα and phosphorylated IκBα to check whether the inhibition of p65 translocation by fermented OYs is related with IκBα degradation. Figure 5B represents both OY and fermented OYs block the phosphorylation of IκBα in the cytosol, implying OYs prevent IκBα degradation and NF-κB activation.

### Effect of OY and fermented OYs on phosphorylation of MAPKs in LPS-stimulated RAW 264.7 cells

**Figure 6:**
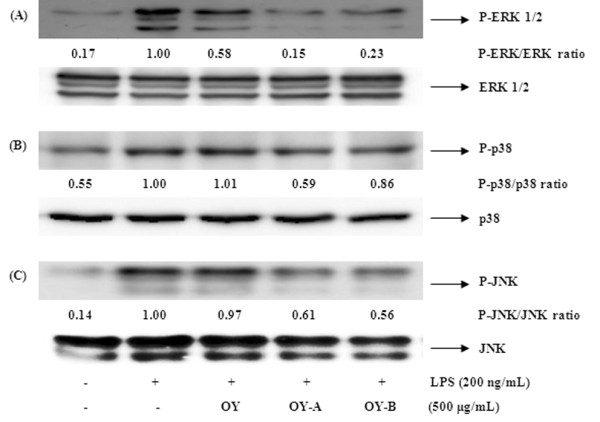
**Effect of OY and fermented OYs on LPS-induced the phosphorylation of MAPKs**. RAW 264.7 cells treated with OY or fermented OYs (500 μg/mL) for 30 min were activated with LPS (200 ng/mL) for 30 min. Whole-cell lysates were prepared for detection of MAP kinases in Western blot analysis.

The MAPKs play a critical role in cell proliferation, differentiation and cellular responses on cytokines or stress inducer. Since MAPK activation by phosphorylation are well-known for NF-κB stimulation [25], we investigated whether the elevated inhibition of NF-κB activation by fermented OYs is mediated by the MAPKs pathway. When we examined the effect of OY and fermented OYs on the phosphorylation of three MAPKs in LPS-stimulated RAW 264.7 cells using Western blot analysis, non-fermented OY showed a little inhibitory effect on phosphorylation of MAPKs, but fermented OYs significantly suppressed all MAPKs phosphorylation at 500 μg/mL LPS stimulation (Figure 6). The amounts of nonphosphorylated MAPKs were not affected by either LPS or OY and fermented OYs treatments. These results indicate fermentation of OY by *Lactobacillus *augments its repressive effect on MAPK activation by LPS.

### High performance liquid chromatography (HPLC) analysis of OY and fermented OYs

**Figure 7:**
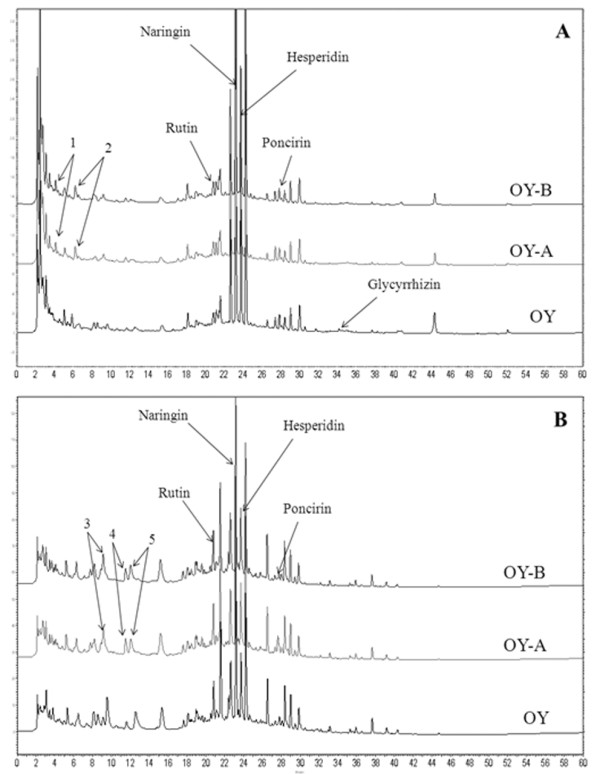
**The HPLC analysis chromatograms of OY, OY-A and OY-B**. (**A**) 280 nm and (**B**) 335 nm; Increased peaks (1-5) were not identified; 1. *t*_R _4.11 min; 2. *t*_R _6.17 min; 3. *t*_R _9.13 min; 4. *t*_R _11.50 min; 5. *t*_R _12.03 min.

**Figure 8:**
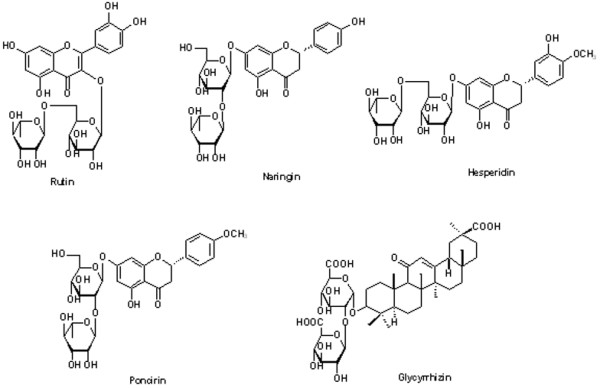
**Chemical structures of five marker constituents in OY, OY-A and OY-B**.

HPLC was used to analyse major marker compounds (1-5) contained in OY, OY-A and OY-B (Figure 7). HPLC analysis chromatograms identified that major constituents of these samples were rutin (*t*_R _20.68 min), naringin (*t*_R _23.20 min), hesperidin (*t*_R _23.74 min), and poncirin (*t*_R _27.81 min) (Figure 8). Glycyrrhizin (*t*_R _34.14 min) was weakly detected in OY, but disappeared in both OY-A and OY-B. Five unknown compounds were increased in the fermented OYs. The structures and identifications of these constituents (1-5) will be elucidated in further study.

## Discussion

Many recent studies on natural herb-derived agents have been investigated to discover the potential anti-inflammatory natural products using *in vitro *and *in vivo *systems. OY is one of important oriental medicine and has been used to treat various diseases such as arthralgia and circulatory disturbance.

Fermentation occurred by microorganism generates various low molecular weight substances from macromolecule like glycoside. Several studies showed fermentation by microorganism is helpful for improving the antioxidant activity of original raw materials [5,9]. In this study, we investigated whether the fermentation by *Lactobacillus *affects the composition of OY and improves anti-inflammatory effect of OY.

**Figure 9:**
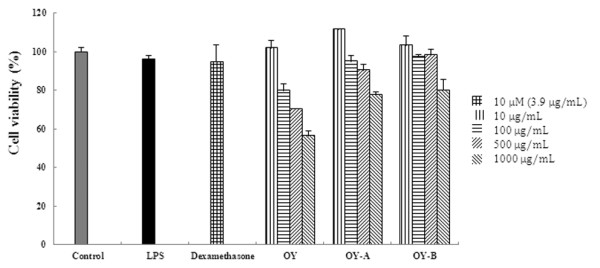
**Assessment of OY and fermented OYs cytotoxicity in RAW 264.7 cells**. The MTT assay was performed after incubation of RAW 264.7 cells treated with different doses (10, 100, 500 and 1000 μg/mL) of OY or fermented OYs for 48 h. Data are mean ± SE value of duplicate determinations from three separate experiments.

Because the overproduction of NO can be harmful and results in various inflammatory and autoimmune diseases [26,27], pharmacological interference of the NO production cascade presents a promising strategy for therapeutic intervention in inflammatory disorders. In this study, we demonstrated that the fermented OYs inhibited more effectively LPS-induced NO and PGE_2 _production than non-fermented OY. To explore the inhibitory mechanism of OY and fermented OYs on NO and PGE_2 _production in RAW 264.7 cells, we examined the effect of OY and fermented OYs on the iNOS and COX-2 genes which are induced in response to inflammatory signals such as cytokines and stimuli [28]. OY showed a little inhibitory effect on COX-2 and iNOS expression, but fermented OY-A and OY-B strongly suppressed COX-2 and iNOS expression in both protein and mRNA levels. These results imply that fermentation of OY increases its inhibitory roles on the expression of pro-inflammatory mediators. When the cytotoxic effect of OY and fermented OYs was evaluated using an MTT assay, OY and fermented OYs did not affect the viability of the RAW 264.7 cells up to concentration of 500 μg/mL (Figure 9). Therefore, we selected 10-500 μg/mL of OY and fermented OYs to examine dose-dependent effect on NO, PGE_2 _and cytokine production. Also, we used OY and fermented OYs at 500 μg/mL for Western blot and RT-PCR analysis.

NF-κB is a major regulatory transcription factor involved in the cellular responses to stimuli such as stress, cytokines, free radicals, ultraviolet irradiation, oxidized LDL, bacterial or viral antigens [29-33]. In addition, it is well known that NF-κB plays an important role in the regulation of cell survival and the expression of pro-inflammatory cytokines [34-38].

Since the expression of these pro-inflammatory mediators is modulated by activated NF-κB, we investigated whether OY or fermented OYs inhibit NF-κB activation. We found that fermented OYs not OY strongly repress both COX-2 and iNOS expression. We also demonstrated that the nuclear translocation of p65 upon LPS stimulation was little inhibited by non-fermented OY, but fermented OYs showed significantly increased inhibition effect on nuclear translocation of p65 through the elevated phosphorylation of IκBα, following to degradation. These findings are consistent with other studies, which found that NF-κB response elements are present on the promoter of the COX-2, iNOS, TNF-α and IL-6 genes [39-42].

There are at least three families of MAPKs (ERK, p38 and JNK MAPKs) which play a critical role in the LPS-induced iNOS expression signaling pathway [43] in mammalian cells. We also checked the effect of OY and fermented OYs on the LPS-induced MAPKs phosphorylation in RAW 264.7 cells. Non-fermented OY showed a little inhibitory effect on the phosphorylation of ERK 1/2 out of MAPKs but the fermented OYs significantly inhibited the levels of phosphorylation of all MAPKs upon LPS stimulation. These findings suggest that the enhanced anti-inflammatory activity of fermented OYs is related with repressed NF-κB and MAPK activity.

## Conclusions

Taken together these results, OY showed a slight inhibitory effect on the LPS-induced production of pro-inflammatory mediators, but OYs fermented by *Lactobacillus *exerted potent anti-inflammatory activity through inhibiting the production of pro-inflammatory mediators including NO, PGE_2_, TNF-α and IL-6 and their synthesis enzymes iNOS and COX-2. Consistent with that, the activities of transcription factor NF-κB and MAPKs related with inflammation were also strongly inhibited by fermented OYs.

In conclusion, OY contains weak anti-inflammatory activity, but OY-A and OY-B fermented by *Lactobacillus *exert remarkably enhanced anti-inflammatory activity than original form on stimulated macrophage cells. Based on the HPLC results, the experiments on which components of OY are modified during fermentation and responsible for the increased activity are in progress. These results suggest the OY-A and OY-B could be developed as a new anti-inflammatory therapeutic herbal medicine without cytotoxicity after further *in vivo *studies.

## Competing interests

The authors declare that they have no competing interests.

## Authors' contributions

YCO, WKC, JHO, GYI, YHJ, MCY and JYM participated in the design of the study, YCO carried out the experiments, analyzed the data and wrote the paper. All authors read and approved the final manuscript.

## Pre-publication history

The pre-publication history for this paper can be accessed here:

http://www.biomedcentral.com/1472-6882/12/17/prepub
